# A randomized controlled trial of cognitive remediation for a national cohort of forensic patients with schizophrenia or schizoaffective disorder

**DOI:** 10.1186/s12888-019-2018-6

**Published:** 2019-01-15

**Authors:** Ken O’Reilly, Gary Donohoe, Danny O’Sullivan, Ciaran Coyle, Aiden Corvin, Padraic O’Flynn, Muireann O’Donnell, Toni Galligan, Paul O’Connell, Harry G. Kennedy

**Affiliations:** 1 Department of Psychiatry Trinity College Dublin, the University of Dublin, Dublin, Ireland; 2 The Central Mental Hospital, National Forensic Mental Health Service Ireland, Dublin, Ireland; 3 School of Psychology, National University of Ireland Galway, Galway, Ireland

**Keywords:** Schizophrenia, Forensic mental health, Cognitive remediation training, CRT, Neurocognition, Effectiveness, Clinical trial

## Abstract

**Background:**

Evidence is accumulating that Cognitive Remediation Training (CRT) is effective for ameliorating cognitive deficits experienced by patients with schizophrenia and accompanying functional impairment. There has been no randomized controlled trial of CRT using a nationally representative population of forensic patients, despite the significant cognitive deficits frequently present within this group.

**Methods:**

Sixty-five patients with schizophrenia or schizoaffective disorder were enrolled in a single blind randomized controlled trial of CRT versus treatment as usual (TAU); representing 94% of those eligible within a national forensic cohort. The primary outcome measure was the composite score of the MATRICS Consensus Cognitive Battery (MCCB). Secondary outcome measures included neurocognitive and social cognitive domains, symptoms, and ‘real world’ functioning. Patient satisfaction was examined using an exit interview. Participants were reassessed at 8 months follow up. All data were analyzed using an intention to treat design (ITT).

**Results:**

For the primary outcome measure, the MCCB composite score, there were significant differences between those who participated in CRT and those receiving TAU at both end of treatment and 8 months follow up (Cohen’s d = 0.34. Significant improvements were observed in visual and working memory. Mediation analysis found that those who cognitively benefited from CRT had corresponding improved functioning, and more net positive therapeutic moves i.e. moves to units with lower security within the hospital. Ninety-six percent believed their cognitive gains positively affected their daily lives.

**Conclusions:**

CRT may be an acceptable and efficacious intervention for forensic patients with schizophrenia or schizoaffective disorder.

**Trial registration:**

ClinicalTrials.gov Identifier: NCT02360813. Trial registered Feb 4th 2015, last updated May 1st 2015.

## Background

Only one in seven patients with schizophrenia achieves functional and symptomatic remission sustained over time [1]. One explanation for the rate of recovery is the degree of cognitive impairment associated with the disorder [2]. Approximately 85% of patients with schizophrenia experience cognitive impairment [3]. The magnitude of cognitive impairment is particularly pronounced when measured using composite scores derived from instruments like the MATRICS Consensus Cognitive Battery (MCCB), which aggregate deficits across cognitive domains affected by the illness [2, 4]. The development of the MCCB has also facilitated direct comparisons of groups of patients regarding the extent of their cognitive impairments [5, 6]. Within a sample of 2616 stable patients participating in North American clinical trials the mean score on the MCCB was approximately 2.5 SD below the nonclinical mean [7]. However, there may be groups of patients who are even more impaired. Forensic patients are detained under mental health legislation with histories of social dysfunction including violence and are often excluded from mainstream research on schizophrenia [8]. Amongst a national cohort of forensic patients, we found that the mean MCCB composite was more than 3 standard deviations (SD) below the nonclinical mean i.e. a level traditionally associated with moderate intellectual disability [6]. In line with systematic reviews of cognitive difficulties experienced by non-forensic patients, the cognitive impairments experienced by forensic patients are also associated with difficulties in ‘real-life’ functioning and impaired ability to benefit from psychosocial treatment programs [9–11]. Addressing the cognitive impairments experienced by forensic patients is therefore an important objective [8].

Cognitive remediation training (CRT) is a behaviorally based treatment for the cognitive deficits associated with schizophrenia. CRT purports to take advantage of ‘neuroplasticity’ through a process of learning known as ‘drill and practice’, in addition to explicitly teaching meta-cognitive strategies [8]. For community patients, there is evidence that CRT is effective for ameliorating cognitive impairment and the associated functional difficulties. A meta-analysis of randomized controlled trials involving 2104 participants found evidence of an effect size (Cohen’s d) of 0.44 on a composite measure of cognition and an effect size of 0.42 for ‘real world’ functioning [12]. There is also evidence that internet delivered cognitive training may be effective for some patients [13]. In keeping with stage-based theories of illness, forensic patients may require different interventions due to the magnitude of their cognitive impairment and social dysfunction, and because of the forensic context in which treatment is offered [8, 14]. However, a modified form of CRT could be particularly useful for this population. Specifically, within a forensic setting CRT may facilitate patients to assume the role of ‘customer’, in line with both research on the importance of goal consensus for the outcome of psychotherapy, and recovery theory [8, 15]. In contrast to many patients’ limited insight into their symptoms and violence risk [16], patients with schizophrenia often have an awareness of their cognitive impairments and are willing to engage in treatment [17]. Following successful completion of CRT forensic patients may be more likely to engage in programs targeting insight, substance misuse, and violence risk.

To date there has been limited investigation of the effectiveness of CRT for forensic patients [8]. Only two randomised controlled trails have been conducted [18, 19]. One study [18] investigated the feasibility of improving social cognition; the second study [19] mixed forensic patients with general mental health patients who were less cognitively impaired. Both trials reported cognitive gains. Neither of these studies adopted the use of a consensus measure of cognitive deficits such as the MCCB. Consequently, it is difficult to estimate the overall degree of cognitive impairment experienced by the participants. It is therefore unknown whether existing studies generalise to forensic patients who may be more severely impaired [8]. This study seeks to address these gaps by testing the efficacy of CRT using a national representative cohort of forensic patients with schizophrenia or schizoaffective disorder. This study may be regarded as an effectiveness study and a robust evaluation of the transportability of CRT to a ‘real-world’ setting [20, 21]. Our model of CRT is outlined in detail within our study protocol and has been specifically developed for forensic patients [8]. We operationalized CRT using nine treatment principles (Table 1). We chose to adopt flexible principles rather than a tightly manualized format in keeping with the common factors model of psychological therapies [8, 15, 22–24] in order to be sensitive to working with forensic patients who have variable levels of ability and unique problems and needs.

**Table 1:** Principles guiding cognitive remediation intervention

Principle 1, Relationship Building: A major focus of each session is to prioritise the development of a strong therapeutic relationship. The therapeutic relationship will be strengthened by providing a credible rational for participation, explicitly linking the cognitive remediation to patients’ goals, promoting success experiences, making participation enjoyable, providing positive reinforcement, and managing ruptures, which may occur during the course of the intervention.	
Principle 2, Collaborative Goal Setting: So as to promote ‘buy in’ patients will be encouraged to develop a series of short term, medium term, and long-term goals. Patients neuropsychological and risk assessments e.g. HCR-20, DUNDRUM toolkit, will be shared with patients to create a platform to develop goals. An explicit connection will also be drawn between cognitive difficulties and patients’ aspirations. Short term goals may include having the concentration required to watch a TV programme or to read a book. Medium term goals may include patients’ ability to self-medicate or move to a less secure unit. Long term goals may include returning to work, and developing relationships outside the hospital.	
Principle 3, Session Structure: Each session will begin with a mood check to establish rapport or identify problems followed by agenda setting, implementation of the agenda items, and summaries before moving to the next agenda item. The session will end by giving patients the opportunity to provide feedback.	
Principle 4, Content of the sessions: The sequencing of interventions will be informed both by patients’ goals and their unique strengths and vulnerabilities as documented by neuropsychological assessment. Cognitive domains at the start of the informational processing stream e.g. attention and vigilance, working memory etc. will typically be prioritised over those occurring later e.g. comprehension and social problem solving. This is because difficulties associated with higher level cognitive processes may be a result of problems with more basic processes such as attention and memory. As patients demonstrate some improvement in core cognitive skills higher level domains will be targeted. Clinical judgement will be required to determine if patients achieve a basic level of mastery in certain cognitive domains or if a ceiling has been reached before progressing to more complex domains. CRT therapists should carefully assess whether patients are improving on core domains e.g. verbal memory etc., and if these improvements are being maintained over time.	
Principle 5, Pacing: Therapists are encouraged to avoid trying to squeeze too much into each session or to work on too many problems simultaneously because it takes time to consolidate skills. In other words, patients need opportunities to repeat tasks again and again to improve performance, which is referred to as massed practice. Throughout the intervention each session should build on the next and be targeted at concrete goals. Patients should be provided with feedback on their progress towards goals. Newly acquired skills should not be abandoned once developed but refreshed during future sessions. Patients may also need breaks between tasks. This down time is a good opportunity to ask patients about their lives and to strengthen the therapeutic relationship.	
Principle 6, Errorless Learning and Scaffolding: Task difficulty should be set so that patients obtain a high level of success on each task to avoid faulty learning and to enhance moral. Patients will be required to obtain a success rate of 80% before the cognitive demands of the task are increased. Where problems are encountered therapists should provide scaffolding and model successful completion of tasks.	
Principle 7, Meta cognitive Strategies: A major focus of each session will be to explicitly teach patients meta-cognitive strategies which are somewhat independent of basic cognitive ability and can be flexibly applied across situations. Examples of meta-cognitive strategies include goal setting, visualisation, focusing on one thing at a time, self-verbalisation, planning, breaking problems into parts, sequencing, chunking, advantage disadvantage analysis, perspective taking, monitoring performance, reflecting on performance etc. It is particularly important to explicitly model the effective use of meta cognitive strategies for patients. The effectiveness of strategies should be carefully assessed using a behavioural experiment framework. The use of particular strategies should be consolidated as evidenced by generalisation before additional meta-cognitive strategies are introduced. When mastery of basic strategies has been consolidated patients can be encouraged to simultaneously use multiple strategies.	
Principle 8 Generalisation: Patients will be encouraged to utilise their cognitive skills outside of remediation sessions by participating in a support group. The focus of the support group will be helping patients to develop a shared understanding of the cognitive deficits associated with schizophrenia, to develop an awareness of how these deficits affect their lives, to identify situations where they can apply their cognitive skills, to obtain encouragement and support from other members of the group on how to implement these skills, to strengthen narratives where success has been achieved. In addition to the above positive group participation in and of itself may enhance cognitive processes as it requires patients to monitor their thoughts, reframe from interruptions, structure their contributions, and reflect on feedback.	
Principle 9 Managing Ambivalence: Patients' ambivalence towards participating should be met in a non-defensive empathic manner. Advantages and disadvantages of participating should be listed using pen and paper to ease the burden on working memory and to model effective problem solving. Patents should be gently reminded of their goals and their initial commitment to participate for the duration of the intervention. Ways of making the cognitive remediation more relevant or enjoyable should be actively explored.	

### Hypotheses

1. That patients allocated to CRT would improve on the primary outcome measure, cognition at the end of treatment, and at 8 months follow up.
2. That patients allocated to CRT would improve on specific neurocognitive and social cognitive domains at end of treatment and 8 months follow up.
3. That patients allocated to CRT would experience improvements in negative and disorganised symptoms.
4. That patients allocated to CRT would experience improvements in real world functioning, net moves to lower level of security, and that patients’ functional improvements or moves to lower levels of security would be mediated by cognitive gains.
5. That patients would experience CRT as a satisfactory and efficacious intervention.

## Methods

### Aim

This study aims to test the efficacy of cognitive remediation training (CRT) using a nationally representative cohort of forensic patients with schizophrenia or schizoaffective disorder.

### Design

This study is a single blind randomized controlled trial of CRT versus treatment as usual (TAU) within a forensic setting.

### Setting

The Republic of Ireland’s National Forensic Mental Health Service (NFMHS) provides care and treatment for adults who have a mental disorder and are at risk of harming themselves or others. At the time of the study the NFMHS had 94 secure inpatient beds located on a single campus (The Central Mental Hospital, CMH). The CMH is the only medium and high secure forensic hospital for the Republic of Ireland, a population of 4.7 million [25].

### Participants

Criteria for inclusion in the trial were being a forensic inpatient with schizophrenia or schizoaffective disorder. The diagnosis of schizophrenia or schizoaffective disorder was established by a consultant psychiatrist using the Structured Clinical Interview for the Diagnostic and Statistical Manual IV (SCID-I) for axis I. Exclusion criteria were: being cared for on an acute unit, lacking capacity to consent, being too dangerous to participate in treatment (positive symptoms combined with aggressive or self-harming behavior in the last month), or being over 65 years of age. Capacity to consent to participation was assessed by the treating consultant psychiatrist. Inclusion criteria were broad and exclusion criteria were minimal because we were primarily interested in investigating the effectiveness of CRT for a nationally representative cohort of forensic patients with schizophrenia or schizoaffective disorder. Sixty-nine patients met inclusion criteria, of whom 65 (95%) provided consent. The Test of Pre-morbid Functioning UK Edition (TOPF-UK) [26] was used in combination with a developmental and educational history. None had a pre-morbid diagnosis of developmental intellectual disability and mean TOPF was within the normal range. All 65 patients who chose to participate had a history of violence and 46% had a history of homicide. Schizophrenia was the diagnosis for 50 (76%) and schizoaffective disorder for 15 (24%). DUNDRUM-1 mean item score was 2.9 (SD 0.46, range 2.2 to 3.8) in keeping with a medium secure level of need [27]. The DUNDRUM-1 triage security instrument is a static assessment of the need for therapeutic security at the time of admission. Socio-demographic and baseline characteristics of all participants are presented in Table 2. Of note, this sample was particularly cognitively impaired with a mean MCCB t-score of 21, 3 SD lower than a nonclinical population mean. The mean Historical Clinical Risk Management version 2 score [28] (HCR-20) for the total sample was 26, SD 5.7.

**Table 2:** Sample characteristics at baseline

	TAU (*N* = 33)		CRT (*N* = 32)		T	df	*p*
	Mean	SD	Mean	SD			
Male/ female	27/6		28/4				
Age	39.30	9.51	42.68	9.74	−1.41	63	0.16
NGRI/ other	18/15		19/13				
CPZeq	472.15	313.52	505.31	362.14	−0.39	63	0.69
ACB	4.09	2.59	3.40	2.49	1.08	63	0.28
MCCB Modified	31.97	9.53	33.19	8.33	−0.54	63	0.58
MCCB	20.30	14.80	22.18	13.61	−0.53	63	0.59
Speed of processing	25.45	14.31	25.65	13.47	−0.05	63	0.95
Working memory	32.09	12.70	32.68	13.96	−0.18	63	0.85
Verbal learning	34.69	7.93	35.71	7.89	−0.52	63	0.60
Visual learning	29.42	12.98	33.65	11.50	−1.38	63	0.17
Problem solving	33.21	8.64	36.43	8.90	−1.48	63	0.14
Social cognition (MSCEIT)	36.96	15.32	35.00	10.93	0.59	63	0.55
Eyes of the mind	20.56	7.62	20.96	5.10	−0.24	54.42	0.80
Faux Pas	38.84	17.25	46.34	11.65	−2.00	54.71	0.05
SOFAS	58.36	13.66	61.06	10.94	−0.87	63	0.38
PANSS Positive symptoms	9.90	5.03	9.65	4.51	0.21	63	0.83
PANSS Negative symptoms	17.09	6.55	15.37	5.54	1.13	63	0.25
PANSS Disorganization	10	4.37	9.68	4.11	0.29	63	0.76
PANSS Excitement	7.30	2.74	7.00	3.6	0.38	63	0.70
PANSS Emotional dysfunction	7.84	2.46	8.53	3.48	−0.91	63	0.36
DUNDRUM-1	2.84	0.52	3.02	0.40	−1.49	59.87	0.14
HCR-20	27.45	5.66	25.12	5.63	1.66	63	0.10

Male/ female Chi square 0.40, df = 1, *p* = 0.52, Age/ gender Chi square 0.15, df = 1, *p* = 0.69

### Randomization and treatment allocation

**Fig. 1:**
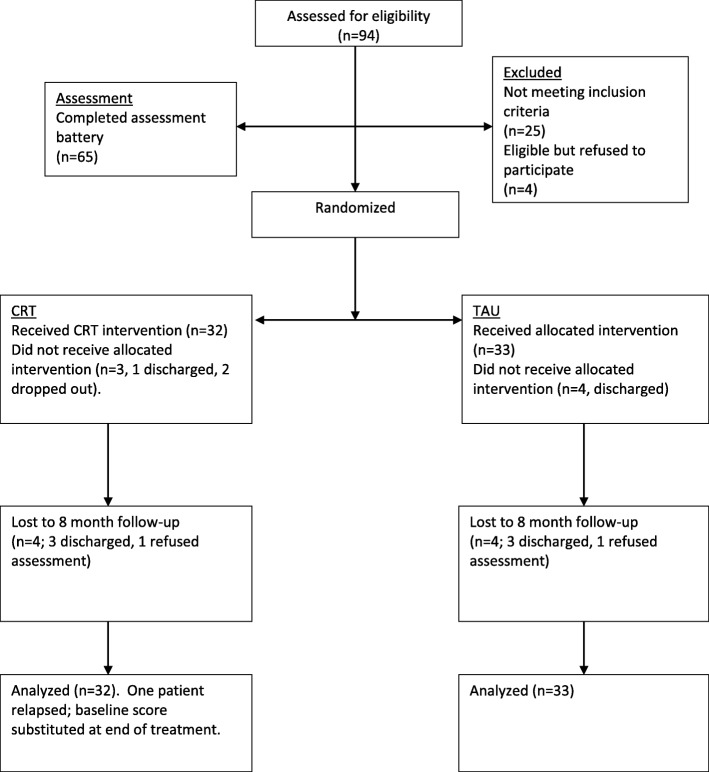
CONSORT flow diagram

Following enrolment participants were randomized using SPSS V21 to CRT or a waiting list control group receiving treatment as usual (TAU). Figure 1 outlines patient allocation (CONSORT diagram). The research team was blinded to group allocation. The clinicians conducting the therapy sessions were different from the research team carrying out the assessments. All patients participating in the study were trained not to reveal their study condition prior to each assessment. Evaluators were tested for ability to ‘see through’ blinding at the end of the study and follow-up. For clinical reasons patient participation in CRT was shared with their treating psychiatrist.

After randomization, 29 (88%) of 33 patients in the control group met criteria for schizophrenia and 4 (12%) for schizoaffective disorder, while in the intervention group 21 (66%) met criteria for schizophrenia and 11 (34%) for schizoaffective disorder. However the two groups did not differ significantly for any measure of neurocognitive or social cognitive ability, symptom severity or functional ability (Table 2). A further sensitivity analysis showed that two groups defined by diagnosis (schizophrenia and schizoaffective disorder) did not differ significantly in any of the variables shown in Table 2.

### Cognitive remediation training

CRT is designed to improve cognitive problems associated with schizophrenia and schizoaffective disorder [12]. Our cognitive remediation training is a principle driven intervention consisting of nine treatment principles, which are flexibly applied during delivery of the intervention ([8]; Table 1). Principle driven approaches are in keeping with the recommendations of a task force on Principles of Therapeutic Change that Work, sponsored by the American Psychological Association and the North American Society for Psychotherapy Research [22] and are also in keeping with a review of effectiveness and common factors [15]. Psychotherapy principle driven approaches integrate research concerning empirically supported treatments (EST) with research concerning the moderating influence of the therapeutic relationship [15, 22, 24]. Patients allocated to CRT received three individual sessions a week and one group session for approximately 14 weeks, 56 sessions in total. Most therapists were masters level psychologists, two therapists were psychiatrists, and another was an occupational therapist. Our CRT program has been extensively described in the study protocol and consisted of a combination of pen and paper and computerized materials [8]. Fidelity to the CRT principles was routinely assessed by randomly observing CRT therapists and by weekly supervision.

### Treatment as usual TAU

Participants in both conditions received TAU from hospital clinicians. At a minimum, this consisted of antipsychotic pharmacotherapy and a therapeutically safe and secure environment appropriate to the individual patient’s needs [11, 25, 27, 29, 30]. The system for delivering ‘treatment as usual’ has been described and shown to be effective in reducing a measure of violence proneness, the HCR-20 [11]. This draws on principles of multi-modal treatment [23] and multi-systemic treatment [24]. Most patients were expected to be involved in a range of therapies. These interventions are organized under seven pillars of care that may be regarded as treatment as usual within a forensic setting: physical health, mental health, drugs and alcohol, problem behaviors, independent living, education-occupation-creativity, and family relationships [27, 30]. Medication was managed by psychiatrists responsible for the patients’ care. Antipsychotic dose CPZeq and anticholinergic burden ACB were measured at each assessment point [8].

### Primary outcome measure: change in global cognitive functioning at end of treatment

Cognitive functioning among study participants was assessed at baseline and end of treatment using the MATRICS Consensus Cognitive Battery (MCCB) global composite score [5]. The MATRICS battery covers seven cognitive domains: processing speed; attention/ vigilance; working memory; verbal learning; visual learning; reasoning and problem solving; and social cognition. Like other cognitive remediation trials, we had trouble with the attentional-vigilance domain of the MCCB which is measured using a Continuous Performance Test [31]. The Continuous Performance Test is administered via computer and because of technical difficulties during the trial we excluded this task from our composite score. Consequently, the MCCB composite was created by averaging the scores over all other domains. In keeping with the recommendations in the MCCB manual we used age and gender corrected scores [5].

### Secondary outcome measures

Cognitive functioning was also assessed at 8 months follow up using the MCCB composite.

### Change in specific cognitive domains

The processing speed, working memory, visual learning, verbal learning, reasoning/ problem solving and social cognitive domains of the MCCB were used as secondary outcome measures.

### Social cognitive measures

Changes in social cognition were assed using the Managing Emotions subtests of the Mayer-Salovey-Caruso Emotional Intelligence Test (MSCEIT) contained within the MCCB [32]. This was supplemented with the Reading the Mind in the Eyes Test [33] and the Faux Pas Recognition Test [34].

### Psychiatric symptoms

A five-factor model of the Positive and Negative Syndrome Scale (PANSS) [35] consisting of positive, negative, disorganized, excitement and emotional dysfunction was used to evaluate outcomes because CRT is thought to have a specific impact on negative and disorganized symptoms [36].

### Real world functioning

The Social and Occupational Functioning Assessment Scale (SOFAS) was used to assess real world functioning [37] (DSM–IV–TR 2000, 4th ed). Higher scores represent superior functioning. The SOFAS was completed by a member of the treating MDT as they were judged best placed to rate the patients functioning.

### Positive moves from more secure to less secure units or discharge to community services

Patients at the CMH are stratified according to level of therapeutic security [29, 38]. Patients are moved from more secure wards to less secure wards and eventually to the community as they progress along the recovery pathway. The placements correspond to levels of risk, symptom severity, and the patient’s overall level of functioning. A positive move represented transfer from a higher to a lower level of security. A negative move represented a transfer from a lower to higher level of security. The net number of positive moves that occurred during the trial was summed for each patient over the duration of the study i.e. at 8 months follow up. For the male patients five positive moves separate the acute unit from living in the community. For female patients who reside on a single ward with acute and stable patients one positive move separates them from the community.

### Patient satisfaction measure

A service-user developed interview for evaluating patient experience of CRT was used to explore patient satisfaction with the intervention [39]. The interview was administered at the end of treatment by a social worker who was independent of treatment and assessment teams and blind to the intervention and to other assessments. Patients were reassured that all responses were anonymously recorded i.e. that their names were not connected with the feedback they provided.

### Statistical analysis

Data analysis was carried out using intention to treat methodology (ITT) [40]. Data from all enrolled participants were used in the analysis regardless of participants’ level of participation in the study using last observation carried forward. The ITT methodology was also utilized at the 8 months follow up to detect whether patients continued to benefit from participating in CRT. All data were analyzed using SPSS V 21. One patient who participated in CRT relapsed at the end of treatment. A decision was made to substitute this patient’s data from baseline for the patient’s end of treatment analysis in keeping with the ITT methodology.

ANOVA and chi-squared tests were used to examine baseline differences between CRT and TAU groups following randomization. At the end of treatment and at 8 months follow up ANCOVAs were carried out in which performance for the outcome of interest i.e. primary and secondary outcomes, was entered as the dependent variable, group (CRT or TAU) as independent variable, and baseline performance on the dependent variable was entered as covariate.

Three mediation analyses were also carried out in line with the study protocol. This was to clarify whether changes in cognition associated with CRT were linked to ‘real world’ functional outcomes including being moved to a unit with a lower level of security i.e. whether changes in cognition were associated with functional change. Mediation analyses were conducted using Hayes’s SPSS PROCESS Macro Model 4 [41]. Bootstrapping (10,000 bootstrap samples) was used with 95% bias corrected confidence intervals applied. In the first mediation analysis, (Table 4 model A) the dichotomous variable CRT vs TAU was the independent variable, real world functioning (SOFAS at end of treatment) was the dependent variable, and neurocognitive functioning (MCCB composite at end of treatment; primary outcome) was the mediating variable. For the second mediation analysis CRT vs TAU was the independent variable, MCCB at 8 months follow up (secondary outcome) was the mediator, and real world functioning (SOFAS at 8 month follow up), was the dependent variable (Table 4, model B). Baseline MCCB and baseline SOFAS were entered as covariates for both analyses. For the third mediation analysis (Table 4 model C) CRT vs TAU was the independent variable, net positive moves over the course of the study i.e. at 8 months follow up, was the dependent variable and neurocognitive functioning (MCCB composite at end of treatment; primary outcome) was the mediation variable to explore the impact that CRT vs TAU had on net positive moves over the course of the study. Because moves occurred throughout the study the MCCB at the end of treatment was entered at the mediator rather than the MCCB at 8 months follow up. Baseline MCCB and gender were entered as covariates for mediation analyses.

## Results

At the end of treatment 29 patients remained in the CRT group (90%) and 28 patients remained in the TAU group (85%). At 8 months follow up 25 patients remained in both groups (78 and 76%). Table 3.

**Table 3:** Outcome measures comparing three time points, baseline, end of treatment period and 8 months follow-up after treatment

Outcome measures	TAU (*N* = 33)						CRT (*N* = 32)						Post			Follow up (8 months)		
	Baseline		Post		Follow up		Baseline		Post		Follow up		F	p	d	F	p	d
	Mean	SD	Mean	SD	Mean	SD	Mean	SD	Mean	SD	Mean	SD						
MCCB modified	31.97	9.53	33.00	9.84	33.14	10.25	33.19	8.33	36.10	8.23	36.35	8.13	4.78	0.03	0.34	4.04	0.04	0.34
Speed of processing	25.45	14.31	26.72	14.11	28.93	17.01	25.65	13.47	28.93	14.17	31.21	14.67	1.49	0.22	0.15	1.41	0.29	0.14
Working memory	32.09	12.70	32	12.9	32.45	13.20	32.68	13.96	36.43	14.5	36.53	12.57	6.67	0.01	0.32	3.90	0.05	0.31
Verbal learning	34.69	7.93	34.33	8.38	35.24	7.98	35.71	7.89	36.53	8.78	38.25	8.22	0.82	0.36	0.25	2.24	0.13	0.37
Visual learning	29.42	12.98	31.63	14.15	30.18	12.26	33.65	11.50	41.25	11.37	37.78	11.91	8.82	0.00	0.79	4.61	0.03	0.62
Problem solving	33.21	8.64	35.03	9.06	34.93	9.15	36.43	8.90	35.75	8.82	36.71	7.98	0.93	0.33	0.08	0.01	0.86	0.20
Social cognition (MSCEIT)	36.96	15.32	38.30	12.81	37.09	14.79	35.00	10.93	37.71	10.77	37.56	10.85	0.06	0.80	0.04	0.95	0.33	0.03
Reading the mind in the eyes test	20.56	7.62	21.59	7.10	21.77	7.68	20.96	5.10	22.36	5.36	22.03	5.06	0.23	0.63	0.12	0.08	0.77	0.01
Faux pas recognition test	38.84	17.25	41.12	14.90	42.48	15.39	46.34	11.65	48.10	12.44	48.62	10.14	0.84	0.36	0.50	0.50	0.48	0.47
SOFAS	58.36	13.66	60.45	14.96	61.81	14.52	61.06	10.94	62.62	12.49	62.03	11.87	0.01	0.90	0.16	0.36	0.54	0.01
Net positive moves	–	–	–	–	.515	.939	–	–	–	–	0.59	0.75	–	–	–	0.10	0.74	0.09
PANSS positive	9.90	5.03	9.42	4.28	9.39	4.77	9.65	4.51	9.15	4.75	8.43	4.92	0.01	0.90	0.05	0.91	0.34	0.19
PANSS Negative	17.09	6.55	15.45	6.09	14.48	6.10	15.37	5.54	15.50	7.24	14.65	7.11	1.34	0.25	0.00	1.03	0.31	0.02
PANSS disorganisation	10.00	4.37	9.24	3.45	9.12	3.28	9.68	4.11	9.03	4.03	9.03	3.76	0.00	0.99	0.05	0.02	0.87	0.02
PANSS excitement	7.30	2.74	6.60	2.96	6.21	2.79	7.00	3.6	7.40	3.61	7.18	3.43	3.29	0.07	0.24	4.09	0.04	0.31
PANSS negative emotions	17.09	6.55	8.18	4.41	7.27	2.41	15.37	5.54	7.78	2.76	7.68	2.65	1.09	0.29	0.10	0.12	0.72	0.16

### Primary cognitive outcome measures at end of treatment, and outcome at 8 months follow up

Differences in MCCB composite scores were compared between the CRT and TAU groups at both end of treatment (primary outcome) and 8-month follow up, using ANCOVAs co-varying for baseline MCCB composite performance. A significant difference in favor of CRT was observed on the MCCB composite at end of treatment (Cohen’s d = 0.34). This difference in favor of CRT for the MCCB composite remained significant at 8 months follow up (Cohen’s d = 0.34) (Table 3).

### Secondary cognitive outcome measures at end of treatment and 8 months follow up

Significant differences were found between CRT and TAU for the MCCB domains of working memory and visual memory at end of treatment; cognitive improvements were not solely attributable to change in the MCCB composite. At 8 months follow up the difference between CRT and TAU was at trend level for working memory, however, the significant difference for visual learning was maintained. There were no significant differences between the other neurocognitive domains at end of treatment or at 8 months follow up (Table 3).

### Social cognitive outcome measures at end of treatment and 8 months follow up

There were no significant differences in the MCCB social cognition task at end of treatment or 8 months follow up. The general cognitive differences that were observed occurred in the absence of any changes in social cognition.

### Symptom measures

There was no significant difference in any of the PANSS factors at end of treatment. At 8 months follow up a significant difference was found in favor of the TAU group for the PANSS excitement factor. There were no significant differences between the CRT and TAU groups for any other PANSS factors.

### Functioning measures

There were no significant overall differences in the SOFAS scores at end of treatment or 8 months follow up, before mediation analysis.

### Positive moves from more secure to less secure units or discharge to community services

There was no overall significant difference before mediation analysis in the number of net positive moves for the CRT group compared to the TAU group at 8 months follow up (Table 3).

### Mediation analyses

In model A, neurocognitive function (MCCB composite at end of treatment) mediated the relationship between CRT and ‘real-world’ functioning (SOFAS) at end of treatment (Table 4) when controlling for baseline MCCB and baseline SOFAS. Improved cognition associated with CRT was associated with improved ‘real world’ functioning. For every one-unit increase in MCCB score associated with participating in CRT there was an increase of 1.60 points on the SOFAS. However, this association did not reach statistical significance at 8 months follow up (Model B) (Table 4).

**Table 4:** Hayes process mediation model 4: regression and mediation coefficients

*n* = 69	Change in Y		C1: direct effect of X on Y before mediation		C2: direct effect of X on Y after mediation		A: indirect effect of X on Y mediated via M		B: direct effect of M on Y adjusted for X	
	*R* ^2^	*p*	Unstandardized effect size	95% CI	Unstandardized effect size	95% CI	Unstandardized effect size	95% CI	Unstandardized effect size	95% CI
Model A										
X = Group M = MCCBT1	0.49	.000	0.49	−4.69, 5.68	−1.10	−6.27, 4.05	**1.60**	**0.12, 4.21**	**0.88**	**0.16, 1.60**
Model B										
X = Group M = MCCBT2	0.45	.000	−1.81	**−**6.83, 3.20	−2.39	**−**7.55, 2.77	0.55	−0.23, 2.63	0.30	**−**0.32, 0.93
Model C										
X = Group M = MCCBT1	0.12	0.04	0.06	**−**0.36, 0.48	−0.08	**−**0.50, 0.33	**0.15**	**0.02, 0.38**	**0.07**	**0.02, 0.13**

Models A and B controlled for baseline MCCB and baseline SOFAS. Model C controlled for baseline MCCB and gender

Model A: Y = SOFAS at end of treatment. Model B: Y = SOFAS at 8 months follow up. Model C: Y = number of net positive moves; Results in bold are statistically significant

*CI* Confidence interval

Participating in CRT and the net number of positive therapeutic moves by end of the study i.e. the 8 months follow up period, was also mediated by neurocognitive function as measured by the MCCB at the end of treatment, when controlling for baseline MCCB and gender (Model C). Those patients who participated in CRT and who benefited cognitively made more positive moves to lower levels of security within the hospital. For every one-unit increase in cognition associated with CRT there was an increase of 0.15 for the number of positive moves through the hospital (Table 4).

### Patient experience of CRT

Twenty-seven of twenty-eight patients who remained in CRT participated in an anonymous interview evaluating patients’ experience of CRT [38]. One refused to participate, and one was discharged. Nearly all reported subjective improvements in cognition (96%) with most feeling the change was maintained at follow up (85%). Twenty-eight percent believed the change would last, 24% said it would change over time, and 24% said that if they did not practice their skills improvements would deteriorate. Ninety-six percent believed the cognitive gains they experienced had positively affected their daily lives. Subjective improvements were noted in a) social interaction, for example decreased interruptions and improving conversational skills; b) engagement in activities, for example participating in other psychosocial treatments; c) working with clinicians, for example remembering the content of multidisciplinary meetings; d) community functioning. Ninety-six percent said that participating had led to positive feelings about themselves and a sense of achievement or confidence. Patients reported that their experience of the relationship with the CRT therapists was important to them (89%). A minority noted aspects that they disliked. Seven mentioned disliking specific tasks (26%). A small number reported anxiety during tasks (7%), some disliked the repetitive nature of sessions (7%). One (4%) disliked the time commitment and tiredness they experienced after sessions. Most said that participating made them aware of their limitations and provided them with insight into their cognitive difficulties (89%). Finally, 26% reported a sense of loss when CRT ended.

## Discussion

The primary aim of this study was to test the effectiveness of CRT within a ‘real world’ population of forensic mental health patients experiencing severe cognitive impairment. The mean score for the forensic patients who enrolled in this study was approximately three standard deviations lower than a nonclinical mean as assessed using the MCCB composite. We were also interested in the acceptability of CRT and patients’ experience of the intervention. Five main outcomes were observed. First, patients who participated in CRT obtained significant improvements in the primary outcome measure, a composite score of the MCCB both at end of treatment and at 8 months follow up. Second, there were significant improvements in specific cognitive domains including working and visual memory, but not social cognition. Third, there were no significant differences in symptoms (PANSS) apart from a difference in favor of the control group in the PANSS excitement factor. Fourth, there were no significant differences between CRT and TAU on routine measures of real world functioning ascertained by the multidisciplinary team (SOFAS) or net positive moves. However, mediation analysis revealed that those who benefited neurocognitively from CRT had related improvement in functioning at the end of treatment (SOFAS); and more net positive therapeutic moves at follow up; there were meaningful functional gains associated with CRT but these gains were predicated on having improved measures of cognitive function. Conversely, those who received CRT but did not have improved cognitive function failed to make ‘real world’ functional gains. Fifth, the patients who were randomly assigned to CRT appeared to value the intervention. Ninety-six percent reported that their subjective neurocognitive ability had improved because of participating in CRT. Importantly 96% percent reported that the cognitive gains they achieved had positively affected their daily lives.

This study contributes to a body of work suggesting that CRT is an effective intervention for patients with schizophrenia or schizoaffective disorder, improving both cognitive and functional outcomes [12, 18, 19]. Although over 40 studies have been conducted, this study overcomes a potential weakness associated with randomized controlled trials namely selection bias [20, 21]. Those who participate in trials may not always be representative of the general population of patients. We believe this study demonstrates ecological validity because of the magnitude of cognitive impairment within the group, and of the 69 patients who met the inclusion criteria in this national service, 65 agreed to take part representing a 94% uptake of those eligible to participate. This study also casts light on the mechanism of action of CRT using mediation analysis. Cognitive improvements associated with CRT were also associated with ‘real world’ functional improvements such as being moving to a unit with a lower level of security. CRT may have the potential to reduce length of stay in secure settings and create savings for services [42].

We controlled for baseline cognition and baseline SOFAS and showed non-the-less that improved cognition associated with CRT was associated with improved real world function (SOFAS). We also showed that when controlling for baseline MCCB and for gender, positive moves were non-the-less associated with improved neurocognition associated with CRT. It may be taken from this that baseline MCCB, SOFAS and gender were not predictors of response to CRT. To clarify the predictors of positive response however would require formal dismantling studies [43, 44].

To date there has only been a small number of RCTs evaluating the effectiveness of psychological interventions within forensic mental health settings [8, 45], and there has been an even smaller number evaluating CRT [18, 19]. This may arise from the misconception that interventions which are efficacious in community settings will be equally effective within forensic settings despite patients being legally detained and potentially more impaired [6, 8]. Forensic services typically have a legally defined dual role requiring care and treatment and in addition public protection [8]. Both roles may not always be aligned and in these cases, it is society and not the patient who is the ‘customer’, which is likely to affect engagement [8]. This study demonstrates that CRT has the potential to improve cognitive functioning for forensic patients in addition to helping patients adopt the role of ‘customer’ [8]. The forensic patients’ response to participating in CRT is particularly striking with the majority of patients regarding the intervention positively. Patients’ positive attitudes towards CRT are likely to be a result of our nine treatment principles, which emphasise the therapeutic relationship, and common factors associated with psychological interventions [15]. CRT may therefore play a useful role by engaging patients to participate in challenging psychological interventions like working on refractory symptoms, violence risk, substance misuse difficulties and pro-social attitudes.

There are limitations and strengths associated with this study. The primary limitation was the numbers of forensic patients available nationally. A robust evaluation of the effectiveness of CRT within forensic services will require a multicentre study involving international collaboration. A strength of this study is that it paves the way for such initiatives. Additional limitations were that medication could not be kept constant during the study, and the absence of an active control group. Additional strengths include having an appropriate dose of therapy [11, 12], the wide range of secondary outcome measures, and the ITT design.

## Conclusion

CRT is an effective intervention for patient groups with schizophrenia experiencing severe cognitive impairments. Those who received CRT demonstrated improved global cognitive performance at the end of treatment and follow up. The high uptake of patients willing to participate, and the positive feedback received suggests that patients’ regarded CRT as an acceptable and valued intervention.

## Abbreviations

95% CI: 95 percent confidence interval
ACB: Anticholinergic burden
ANOVA: Analysis of variance
CMH: Central Mental Hospital
CONSORT: Consolidated Standards of Reporting Trials
CPZeq: Chlorpromazine equivalent
CRT: Cognitive remediation training
DUNDRUM-1: Dangerousness, Understanding, Recovery and Urgency Manual (The Dundrum Quartet)
HCR-20: Historical Clinical Risk Management, Version 2
ITT: Intention to treat
MCCB: MATRICS Consensus Cognitive Battery
MSCEIT: Mayer-Salovey-Caruso Emotional Intelligence Test
NFMHS: National Forensic Mental Health Service
PANSS: Positive and negative syndrome scale
SCID: Structured Clinical Interview for the Diagnostic and Statistical Manual IV (SCID)
SD: Standard deviation
SOFAS: The social and occupational functioning assessment scale
SPSS: Statistical package for the social sciences
TAU: Treatment as usual
